# Expressed sequence tags from *Atta laevigata *and identification of candidate genes for the control of pest leaf-cutting ants

**DOI:** 10.1186/1756-0500-4-203

**Published:** 2011-06-17

**Authors:** Cynara M Rodovalho, Milene Ferro, Fernando PP Fonseca, Erik A Antonio, Ivan R Guilherme, Flávio Henrique-Silva, Maurício Bacci

**Affiliations:** 1 Center for the Study of Social Insects. Univ. Estadual Paulista, Av. 24A, 1515, Bela Vista, Rio Claro, São Paulo, Brazil; 2 Department of Genetics and Evolution, Federal University of São Carlos, Via Washington Luis, Km 235, CP 676, São Carlos, São Paulo, Brazil; 3 Department of Computer Science, Federal University of São Carlos, Via Washington Luis, Km 235, CP 676, São Carlos, São Paulo, Brazil; 4 Department of Statistics, Applied Mathematics and Computation. Univ. Estadual Paulista, Av. 24A, 1515, Bela Vista, Rio Claro, São Paulo, Brazil

## Abstract

**Background:**

Leafcutters are the highest evolved within Neotropical ants in the tribe Attini and model systems for studying caste formation, labor division and symbiosis with microorganisms. Some species of leafcutters are agricultural pests controlled by chemicals which affect other animals and accumulate in the environment. Aiming to provide genetic basis for the study of leafcutters and for the development of more specific and environmentally friendly methods for the control of pest leafcutters, we generated expressed sequence tag data from *Atta laevigata*, one of the pest ants with broad geographic distribution in South America.

**Results:**

The analysis of the expressed sequence tags allowed us to characterize 2,006 unique sequences in *Atta laevigata*. Sixteen of these genes had a high number of transcripts and are likely positively selected for high level of gene expression, being responsible for three basic biological functions: energy conservation through redox reactions in mitochondria; cytoskeleton and muscle structuring; regulation of gene expression and metabolism. Based on leafcutters lifestyle and reports of genes involved in key processes of other social insects, we identified 146 sequences potential targets for controlling pest leafcutters. The targets are responsible for antixenobiosis, development and longevity, immunity, resistance to pathogens, pheromone function, cell signaling, behavior, polysaccharide metabolism and arginine kynase activity.

**Conclusion:**

The generation and analysis of expressed sequence tags from *Atta laevigata *have provided important genetic basis for future studies on the biology of leaf-cutting ants and may contribute to the development of a more specific and environmentally friendly method for the control of agricultural pest leafcutters.

## Background

The tribe Attini comprises over 200 ant species [1] which culture mutualistic fungi for their feeding [2]. The most evolutionary derived attines are the leaf-cutting ants in the genera *Atta *and *Acromyrmex *which are considered major herbivores in the tropics [3].

Some *Atta *species contributes to nutrient cycling, aeration and drainage of water in the soil [4], as well as maintenance of plant diversity [5,6]. Their nests were also found to host arthropods [7-9], reptiles and amphibians [4], and microorganisms [10-14].

However, despite of these ecological roles, many leafcutter species are considered agricultural pests which impose severe economic damages to agriculture [15,16]. Some of the characteristics contributing to the pest status of leafcutters are their ability of exploiting a great variety of plant species [17], reaching high population density [15] and long life spanning queens constantly laying eggs for up to 15 years [18].

*Atta laevigata *is a pest leafcutter distinguished by a very large and shiny head in soldiers, a characteristic which has rendered the species with the popular name "cabeça de vidro" (meaning glass head). It can be found in Venezuela, Colombia, Guyana, Bolivia, Paraguay and, in Brazil, from the Amazonian Rain Forest in the North to the Paraná state in the South [19]. It cuts leaves from many plantations, like pine tree [20], cocoa [21] and eucalyptus [22], as well as wide variety of native plants from different biomes such as the Cerrado or the Rain Forest, where its intense herbivory challenges reforestation of degraded areas [23,24].

The control of pest leafcutters in small properties can be done by biological methods [25] or even utilizing the waste material generated by the ants [26], but in extensive monocultures this control utilizes massive amounts of broad spectrum insecticides which are toxic to other animals and persist in the environment [27]. Thus, the development of a more specific and environmentally friendly process for controlling the leafcutters is required [28].

Genomic studies can contribute with that by characterizing genes involved in key functions for the leafcutters, like longevity, fertility and plasticity to exploit different vegetations, raising more specific targets for the ant control. Genomics is also a valuable resource for ecological and evolutionary studies of leaf-cutting ants.

In the present investigation, we carried out a genomic study in the pest leafcutter *Atta laevigata *by generating 3,203 expressed sequence tags (ESTs) which characterized 2,006 unique sequences (US). We postulate important differences in expression level among the transcripts and identified 146 potential target sequences for the control of pest leaf-cutting ants.

## Methods

### EST generation

Two grams of soldiers and major workers of *Atta laevigata *were macerated under liquid nitrogen, total RNA was extracted with the TRIzol method (Invitrogen, UK) and mRNA was purified using the PolyATract System (Promega, USA). The CloneMiner cDNA Library Construction Kit (Invitrogen, UK) and 2 μg of mRNA were utilized for the synthesis of first and second cDNA strands which were then size-fractioned in a 1.0 ml Sephacryl S-500 resin column, inserted in a pDONR222 plasmid (Invitrogen, UK) and transformed into DH10B *Escherichia coli*. Cells were plated onto solid Circle Grow medium (QBIO-GENE, Canada) containing 25 μg.ml^-1 ^kanamicin and individually picked into a permanent culture plate with 96 wells. After 22 hours growth in liquid Circle Grow medium (25 mg.ml^-1 ^kanamicin), plasmid DNA was purified by alkaline lysis [29] and sequenced in reactions containing 300 ng template DNA, 5 pmol M13 forward primer and the DYEnamic ET Dye Terminator kit reactant (GE Healthcare, UK), according to the manufacturer's protocol. The amplified products were resolved in a MEGA-BACE 1000 automated DNA sequence machine (GE Healthcare, UK).

### EST analysis

The pipeline generation system EGene [30] was used to clean and assemble ESTs in contigs and singlets. Sequences were filtered by quality using phred values >20 and 90% of minimum identity percent in window. Filtered sequences were then masked against vector and primer sequences, selected by size (>100 bp) and assembled using CAP3 [31] with an *overlap percent identity cutoff *(p) of 90 and a *minimum overlap length cutoff *(o) of 50.

The program Blast2GO (B2G) [32] was used to associate every *Atta laevigata *singlet and contig to blastx [33] results (nr protein database; E-value ≤ 10^-5^), Gene Ontology (GO) terms [34], InterProScan classification [35,36] including signal peptide [37] and transmembrane regions predictions, Kyoto Encyclopedia of Genes and Genomes (KEGG) maps (http://www.genome.jp/kegg/), and Enzyme Commission (EC) numbers (IUBMB). The results generated by B2G and those obtained from Conserved Domain Database (CCD) were manually inspected, in order to group contigs and singlets in functional categories and to infer transcript abundance in *Atta laevigata*.

## Results and Discussion

### EST generation and assembly

The 5' ends of 4,704 clones from the *Atta laevigata *cDNA library were sequenced, resulting 4,482 reads. We were able to selected 3,203 of these reads, which presented high-quality and with average length of 418 bp (Table 1; [GenBank:JG659458 to JG662660, dbEST ID:73713535 to 73716737, Genome Project ID:63563]).

**Table 1:** EST processing.

Sequence	Number	%
**Reads**	**4,482**	**100.00**
Filtered by quality	1,241	27.69
Filtered by size	38	0.85
High-quality (after filtering)	3,203	71.46
Unique Sequences (US)	**2,006**	**100.00**
Singlets	1,666	83.05
Contigs*	340	16.95

*Contigs were composed by 1,537 reads.

The high-quality sequences were assembled in 340 contigs (619 bp average) and 1,666 singlets which we assume to represent 2,006 unique sequences (US). It is likely that some of the US came from the same gene due to non-overlapping ESTs from a single gene or products of alternative splicing [38].

### Comparative analysis of *Atta laevigata *genes

**Figure 1:**
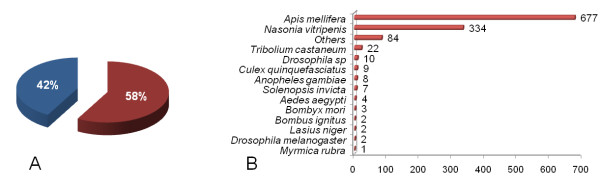
**Summary of Blastx search results for *Atta laevigata *sequences**. (A) Percent of *Atta laevigata *sequences with significant matches (red, Blastx E-value ≤ 10^-5^) and non-significant matches (blue) in the GenBank. (B) Number of best hits found in different biological species.

Using Blastx we found that 1,165 (58%) of the characterized *Atta laevigata *US matched significantly (E-value ≤ 10^-5^) with GenBank sequences in the non-redundant (nr) database (Figure 1A). Most of the best hits (Figure 1B) came from the hymenopterans *Apis mellifera *[39] (677) and *Nasonia vitripenis *[40] (334) genomes, but only 10 hits came from the ants *Solenopsis invicta*, *Lasius niger *or *Myrmica rubra *because ant sequences are relatively poorly represented in the nr database.

**Figure 2:**
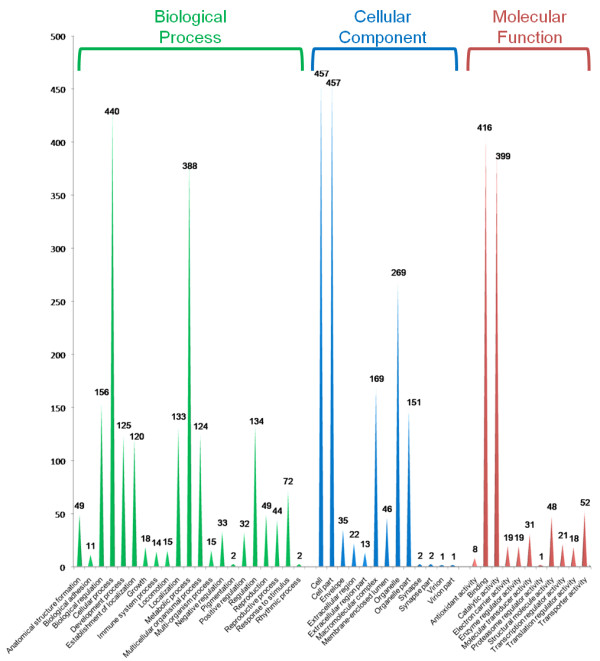
**Distribution of GO terms**. The graphic displays the GO terms at level 2 for each category.

**Figure 3:**
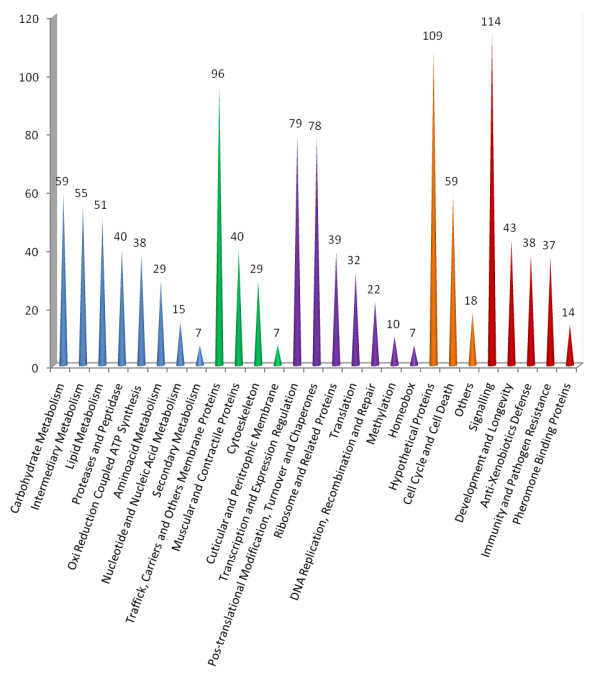
**Functional classification of *Atta laevigata *genes**. The graphic displays the 1,165 US grouped in 27 functional categories. The colors represent major functions: blue: metabolism; green: structural; purple: regulation; orange: other; red: control candidates.

We used B2G program and found GO terms (Figure 2) to 865, EC numbers to 250, predicted signal peptides in 229, and domain information for 66 *Atta laevigata *US, as well as KEGG information. This bulk of retrieved information and data obtained from CDD were manually inspected to annotate *Atta laevigata *US in 27 functional categories (Figure 3).

The number of US per category gives us an idea on the diversity of genes existing in each cell function. This diversity was found high within transcripts related with signaling pathways, membrane or regulation of gene expression, but very low within transcripts related to secondary metabolism, cuticular and peritrophic membranes or homeobox.

### Variation of the number of reads per contig

**Figure 4:**
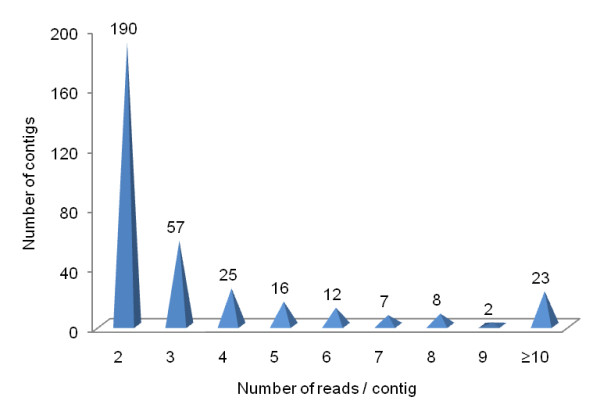
**Contig amount as function of number of reads per contig**. The graphic shows the number of reads used in the assembly of the 340 contigs.

The number of reads per contig varied from two to 123, with 73% of the contigs containing two or three reads and only 7% containing 10 or more reads (Figure 4). Therefore few contigs concentrated many reads, i.e. 1.1% (23 out of 2,006) of the contigs contained 18.8% (603 out of 3,203) of the reads. By dividing the number of reads (3,203) by the number of US (2,006) it was found the average of 1.6 reads per contig. Some of the contigs exceeding this average value are shown in Table 2. Whether the number of reads per contig is related to gene expression level, it can be assumed that *Atta laevigata *contains a set of 23 highly expressed genes. Sixteen of these genes are involved with three major cellular processes (Table 2): (i) ATP synthesis coupled to redox reactions in mitochondria (273 reads); (ii) muscle or cytoskeleton structure (135 reads); (iii) transcription regulatory processes through homeobox or signaling proteins (95 reads). Gene expression is energetically expensive and is accompanied by protein synthesis for the translational process which is even more expensive. The increasing of the number of transcripts of a given gene, even in a very small extent, is not a neutral process but rather strongly constrained by evolution [41], and expected to occur only if positively selected. Therefore, our results suggest that high expression levels have been positively selected in *Atta laevigata *for genes responsible for energy conservation, cell structure and regulation.

**Table 2:** Contigs with high read number in the *Atta laevigata *cDNA library.

Contig	Reads	Rate*	Best hit (organism)	**Function**^**+**^
311	123	76.9	COX I (*Myrmica rubra*)	1
235	52	32.5	Similar to paramyosin CG5939-PA (*Apis mellifera*)	2
183	47	29.4	ATP synthase F0 subunit 6 (*Camponotus sayi*)	1
037	43	26.9	COX III (*Bombyx mandarina*)	1
294	40	25.0	Similar to muscle protein 20 CG4696-PA (*Apis mellifera*)	2
056	30	18.8	COX II (*Atta colombica*)	1
330	30	18.8	Actin-5 (*Bactrocera dorsalis*)	2
289	25	15.6	Similar to limpet CG32171-PD (*Apis mellifera*)	3
337	20	12.5	Muscle LIM protein (*Nasonia vitripennis*)	3
273	19	11.9	Cytochrome b (*Formica pratensis*)	1
268	14	8.8	Similar to muscle LIM protein at 84B (*Apis mellifera*)	3
046	14	8.8	Similar to CG5023-PA (*Apis mellifera*)	3
015	13	8.1	Troponin I (*Apis mellifera*)	2
116	12	7.5	Similar to muscle LIM protein at 84B (*Apis mellifera*)	3
069	11	6.9	NADH dehydrogenase subunit 4 (*Harpiosquilla harpax*)	1
328	10	6.3	AGAP005400-PA (*Anopheles gambiae*)	3

* Number of reads divided per 1.6 which is the mean number of reads (3,203) per US (2,006).

^**+**^**1**. ATP synthesis coupled to redox reactions in mitochondria (273 reads). **2**. Muscle or cytoskeleton structure (135 reads). **3**. Transcription regulatory processes through homeobox or signaling proteins (95 reads).

### Identification of candidate genes for the control of pest leafcutters

Inhibition of the translation of genes which play essential functions in insects by feeding these insects with dsRNA [42] or using transgenic plants [43] seems a promising procedure for the control of agricultural pests [44]. One of the advantages of this procedure is that it targets mRNA molecules which may be species-specific.

In order to control pest leafcutters by inhibiting gene translation, one needs to identify and sequence target candidate genes. Our library was found to contain 146 US which represent potential target genes for the control of leafcutters, because these US are likely playing essential functions in *Atta laevigata *(Table 3). These target genes are related to antixenobiosis (including insecticide detoxification), queen longevity, larval development, insect immunity or resistance to pathogens, communication necessary to social tasks, polysaccharide metabolism or insecticide action.

**Table 3:** Candidate genes for the control of pest leafcutter ants.

Process [GenBank Acc*]	Description	US
Antixenobiosis	Cytochrome P450 activity	25
[JI332418-JI332429, JI332686-JI332710]	Cell detoxification	12
Development and longevity	Development, growth and differentiation	18
[JI332430- JI332440, JI332711-JI332736]	Oxidative stress protection	13
	Juvenile hormone binding and synthesis	6
Immunity and resistance to pathogens	Immune response	29
[JI332441-JI332452, JI332737-JI332761]	Serine protease inhibitor	4
	Melanization and pathogen encapsulation	4
Communication [JI332453-JI332457, JI332762-JI332767]	Pheromone/odorant binding and transport	11
Signaling	Generation and stability of signaling	6
[JI332458, JI332459, JI332768-JI332773]	Acetylcholine receptor	2
Behavior	Courtship and behavior	5
[JI332460, JI332461, JI332774-JI332782]	Learning and memory	3
	Others	3
Polysaccharide metabolism [JI332462, JI332463, JI332783]	Glycogen and starch degradation	3
Intermediary metabolism [JI332464, JI332465]	Arginine kynase activity	2

**Total**		146

*Transcriptome Shotgun Assembly database.

The function and potential utilization of these 146 US as targets for the control of pest leafcutters are considered below.

### Antixenobiotic genes

Cytochrome P450, carboxylesterases, and glutathione transferases are involved in insecticide metabolism [45]. In insects, P450 also participates in the metabolism of many endogenous (including juvenile hormones, ecdysteroids, and pheromones) and exogenous compounds (plant allelochemicals and insecticides) [46].

The enzyme glutathione S-transferase catalyzes the initial conjugation of insecticides with glutathione. Both enzyme and glutathione are very abundant in the cells and essential for detoxification of electrophiles causing cytotoxic or genotoxic damage [47]. The enzyme may play a role in insecticide resistance [48], herbicide resistance in plants [49], resistance of cancer cells to chemotherapeutic agents [50], and antibiotic resistance in bacteria [51]. In our study we found 25 US in the *cytochrome P450 *family and 12 US probably related with detoxification of xenobiotics, including *glutathione S-transferase*, *glutamate cysteine ligase *and *aldehyde oxidase *(Table 3). All these genes may be important targets for the control of leafcutters.

### Development and longevity genes

Of the 18 US we found (Table 3) involved with development, growth and differentiation, four are putatively related with nervous system development, two of which contained the immunoglobulin domain: one *wrapper *one *lachesin *homolog. The protein *lachesin *has a role in early neuronal differentiation as well in axon outgrowth, cell recognition events, cell adhesion or intercellular communication [52]. The other 14 US in this category (Table 3) may be involved in different phases of insect development like egg, or larvae, or development of tissues or organs like mesoderma, spermatechae and antennae.

Queen and worker ants develop from identical eggs, being genetically identical, but the caste system produces a long-lived queen and a short-lived worker with up to ten-fold lifespan differences [3]. Harman [53] stated that lifespan is determined by the rate at which oxidative damage occurs due to the accumulation of by-products of oxidative energy metabolism. Harman's theory implicates that long-lived organisms produce fewer reactive oxygen species or have increased antioxidant production [54], although the degree of lifespan extension can be sex- or genotype-specific [55] and sometimes poorly correlated with antioxidant levels [56].

We found 13 US likely involved in organism lifespan by protection from oxidative stress (Table 3) and which are directly involved in the degradation of superoxide radicals and hydrogen peroxide or neutralization of reactive oxygen species, such as the putative *Cu/Zn superoxide dismutase*, *catalase*, *Rpd3 histone deacetylase*, *peroxiredoxin 5*, *thioredoxin reductase *and *phospholipid hydroperoxide glutathione peroxidase*.

Our library contained four US putatively coding for juvenile hormone binding protein (JHBP) domain and two for putative proteins that participate in JHBP biosynthesis (Table 3). Juvenile hormones (JH) regulate a great number of physiological processes in insect development. Larvae requires JH to maintain larval state and JH must be absent in the last larval instar for metamorphosis to start [57,58]. They are also necessary for reproduction in adults [59].

The characterization of genes which are related to development and longevity in *Atta laevigata *allows future investigation on the effect of the expression of these genes on queen maturation and lifespan, which are a key features associated with leafcutter pest ability.

### Genes associated with immunity and resistance to pathogens

Pathogens, parasites or injury triggers in insects innate immune responses that are in essence similar and comprise both cellular and humoral components. Cellular mechanisms include phagocytosis by special blood cells and encapsulation of large invaders [60]. Humoral responses involve events of proteolytic cascades leading to melanization [60] and the production of antimicrobial peptides initiated via two distinct signaling pathways, Toll and Immune Deficiency, which depend on the pathogen recognition [61]. There are two types of recognition proteins: peptidoglycan recognition proteins and Gram-negative bacteria-binding proteins.

We found 37 US that may be involved with immunity or pathogen resistance (Table 3), including the putative *toll like interacting protein*, *prophenoloxidase subunit 3 *and *easter *CG4920-PA, the last two with role in melanin synthesis. We also found sequences putatively coding for the antimicrobial peptides *hymenoptaecin *and *defensin 2*, and for the *peptidoglycan recognition protein precursor, *as well as *transferrin *and *transferrin 2 *which participate in response to microbial infection by sequestering iron that is an essential nutrient for some pathogens [62].

Leaf-cutting ants and their mutualistic fungus are constantly challenged by pathogenic microorganisms [63] which ultimately regulate host population [64]. Therefore, the 37 US we found probably involved in resistance to microbial pathogens are important markers for understanding antimicrobial mechanisms in leafcutters and putative targets for controlling pest leafcutters.

### Communication genes

Communication plays a central part in social insects necessary for division of labor and task partitioning which are essential for harvesting food, nursing the broods and sexual reproduction [65]. Thus, targeting genes involved in communication seems a promising strategy for the control of leaf-cutting ants.

Our library contained 11 US probably related to communication, one of them putatively coding for the pheromone binding protein (PBP), which is important for chemical recognition of insect conspecifics by transporting odorant molecules from cuticular pores to receptors [66]. In *Solenopsis invicta*, the gene *Gp-9*, which is a PBP homolog, seems to have a role in worker ability to discriminate queens and regulate their numbers [67]. Other important communication gene found putatively codes for fatty acid binding protein involved in transport of communication molecules in insects [68].

Four of the communication US we found were in the lipocalin family which is composed of secreted proteins binding small hydrophobic molecules or forming macromolecular complexes associated with cell surface receptors important for transport, pheromone signaling and olfaction [69]. These sequences putatively code for the odorant binding proteins, apolipophorin III or PP238.

We also found three homologs to the chemosensory protein from *Nasonia vitripennis*, chemosensory protein 2 from *Apis mellifera *and chemosensory protein 5 from *Bombyx mori*. Chemosensory proteins may be specifically expressed in sensory organs which are important in ant behavior [70] and participate in cellular processes that require lipophilic compounds [71].

The putative genes *gustatory receptor *and *dihidrooratate dehydrogenase *involved in odorant reception in insects were also found.

### Signaling genes

Tetraspanin is an important signaling membrane protein expressed in antennae of moths and honeybees [72], being a molecular facilitator of signal transduction and cell adhesion [73]. In our library, six US putatively coding for tetraspanin were present.

We also found two US corresponding to nicotinic acetylcholine receptor which plays a role in visual processing, learning and memory, olfactory signal processing, and mechanosensory antennal input in honeybee [74]. These receptors are targets of neonicotinoids insecticides used against piercing-sucking pests [75].

### Behavior genes

Eleven *Atta laevigata *US in this category (Table 3) were homolog to genes involved in behavior, learning, memory and courtship in *Apis mellifera*, *Drosophila melanogaster *or *Solenopsis invicta*. Some of the genes controlling social behavior and complex tasks or abilities may be specific to Hymenoptera [38] and thus may be specific targets for the control of pest leafcutters.

### Polysaccharide metabolism genes

Food sources for worker leafcutters relies mostly on the plant polysaccharides cellulose, xylane and starch, which are degraded by extracellular enzymes secreted by the mutualistic fungus [76], generating mono and disaccharides readily assimilated by the ants [77]. Degradation of cellulose by the mutualistic fungus generates cellobiose [10] and degradation of starch generates maltose, both disaccharides being consumed by leafcutters [77] through the production of alpha- and beta-glucosidase, respectively. In addition, workers assimilate starch at certain extent [77], which demands production of alpha-amylase.

Our library contained 59 US (Figure 3) corresponding to genes related to carbohydrate metabolism, including *alpha-glucosidase-like*, *beta-glucosidase *and *alpha-amylase *(Table 3) which are promising targets for leafcutters control.

### Arginine kinase gene

Arginine kinase catalyses the reversible transfer of phosphate between ATP and guanidine substrates and acts in cells that need readily available energy sources [78]. This enzyme activity in cockroaches was found to be inhibited by nitrates and borates [79] which were then used as insecticides. Our library contained two US which are putative *arginine kinase *genes (Table 3) that may also be important for the control of leafcutters.

### Future perspectives

The 146 US here proposed as targets for the control of leaf-cutting ants can be used for primer designing in order to study gene expression through real time PCR. For instance, over-expression of sequences here proposed as related to immunity or antixenobiosis in *A. laevigata *challenged by pathogens or insecticides should validate the protective role of the respective gene products in leafcutters exposed to adverse conditions, helping us to understand the molecular basis of pest ant resistance to hazardous chemicals. A future scenario can be envisaged in which inhibition of gene expression, gene translation or the related protein activities would make pest leafcutters more susceptible to pathogens, insecticides or anti-herbivory chemicals produced by crops. In summary, inhibition of genes or gene products related to the processes described in Table 3 may specifically hamper the colonization of crop areas by pest leafcutters.

## Conclusion

Leaf-cutting ants are the major neotropical herbivores, many of which are important agricultural pests. We characterized 2,006 unique sequences (US) in *Atta laevigata*, one of the most geographically spread pest leaf-cutting ant in South America, and found that 16 of the genes are likely under positively selected high expression and responsible for energy conservation or cell structuring or regulation. Another set of 146 US which play important part in anti-xenobiosis, longevity, immunity, development, communication, nutrition or insecticide action were identified as putative targets for the control of pest leafcutters. Our findings provided genetic background for basic and applied studies on these ants.

## List of abbreviation used

**EST**: Expressed Sequence Tags; **US**: unique sequences; **mRNA**: messenger RNA; **cDNA**: complementary DNA; **bp**: base pair; **B2G**: Blast2GO; **nr**: non-redundant; **GO**: Gene Ontology; **KEGG**: Kyoto Encyclopedia of Genes and Genomes; **EC**: Enzyme Comission; **IUBMB**: International Union of Biochemistry and Molecular Biology; **CDD**: Conserved Domain Database; **JHBP**: juvenile hormone binding protein; **JH**: juvenile hormone; **PBP**: pheromone binding protein; **PCR**: polymerase chain reaction.

## Competing interests

The authors declare that they have no competing interests

## Authors' contributions

Conceiving and designing of the experiments: CMR, MB. Construction and sequencing of the cDNA library: CMR, FFPP, FHS. Bioinformatic analyses: MF, EAA, IRG. Contribution with reagents, materials and analysis tools: FHS, MB. Manual annotation and manuscript preparation: CMR, MF, MB. All authors read and approved the final manuscript.

## References

[B1] Bolton BA Alpert G Ward PS Naskrecki P Bolton's catalogue of ants of the world: 1758-2005 2006 Cambridge: Harvard University Press21697660

[B2] Mueller UG Schultz TR Currie CR Adams RM Malloch D The origin of the attine ant-fungus mutualism Q Rev Biol 2001 76 169 97 10.1086/393867 11409051

[B3] Hölldobler B Wilson EO The Ants 1990 Massachusetts: Belknap Press of Harvard University

[B4] Weber NA Gardening ants: the Attines 1972 Philadelphia: The American Philosophical Society

[B5] Garrettson M Stetzel JF Halpern BS Hearn DJ Lucey BT McKone MJ Diversity and abundance of understorey plants on active and abandoned nests of leaf-cutting ants (*Atta cephalotes*) in a Costa Rican rain forest J Trop Ecol 1998 14 17 26 10.1017/S0266467498000029

[B6] Wirth R Herz H Ryel RJ Beyschlag W Hölldobler B Herbivory of leaf-cutting ants. A case study on Atta colombica in the tropical rain forest of Panama 2003 Berlin: Springer

[B7] Moser JC Neff SE *Pholeomyia comans *(Diptera: Milichiidae) an associate of *Atta texana*: larval anatomy and notes on biology Z Angew Entomol 1971 69 343 348

[B8] Steiner WE The first records of *Bycrea villosa *Pascoe (Coleoptera: Tenebrionidae) in the United States, Central America and Colombia and notes on its association with leaf-cutting ants Coleopterists Bulletin 2004 58 329 334 10.1649/619

[B9] Waller DA Moser JC Vander Meer RK, Jaffe K, Cedeño A. Boulder Invertebrate enemies and nest associates of the leaf cutting ant *Atta texana *(Buckley) (Formicidae, Attini) Applied myrmecology: a world perspective 1990 Colorado: Westview Press 255 273

[B10] Bacci M Anversa MM Pagnocca FC Cellulose degradation by Leucocoprinus gongylophorus, the fungus cultured by the leaf-cutting ant *Atta sexdens rubropilosa* Antonie van Leeuwenhoek Int J Gen Mol Microbiol 1995 67 385 386 10.1007/BF00872939 7574556

[B11] Carreiro SC Pagnocca FC Bacci M Lachance MA Bueno OC Hebling MJA Ruivo CCC Rosa CA *Sympodiomyces attinorum *sp nov., a yeast species associated with nests of the leaf-cutting ant *Atta sexdens* Int J Syst Evol Microbiol 2004 54 1891 1894 10.1099/ijs.0.63200-0 15388759

[B12] Currie CR A community of ants, fungi, and bacteria: A multilateral approach to studying symbiosis Annu Rev Microbiol 2001 55 357 380 10.1146/annurev.micro.55.1.357 11544360

[B13] Rodrigues A Pagnocca FC Bacci M Hebling MJA Bueno OC Pfenning LH Variability of non-mutualistic filamentous fungi associated with *Atta sexdens **rubropilosa *nests Folia Microbiol 2005 50 421 425 10.1007/BF02931424 16475502

[B14] Pinto-Tomás AA Anderson MA Suen G Stevenson DM Chu FST Cleland WW Weimer PJ Currie CR Symbiotic nitrogen fixation in the fungus gardens of leaf-cutter ants Science 2009 326 1120 1123 10.1126/science.1173036 19965433

[B15] Fowler HG Silva VP Saes NB Lofgren CS, Vander Meer RK Population dynamics of leaf-cutting ants: a brief review Fire ants and leaf-cutting ants: biology and management 1986 Boulder, Colorado: West-View Press 123 145

[B16] Cameron RS Distribution, impact and control of the Texas leaf-cutting ant: 1983 survey results 1985 Texas Forest Service Publication

[B17] Vasconcelos HL Foraging activity of two species of leaf-cutting ants (Atta) in a primary Forest of the Central Amazon Insectes Soc 1990 37 131 145 10.1007/BF02224026

[B18] Keller L Queen lifespan and colony characteristics in ants and termites Insectes Soc 1998 45 235 246 10.1007/s000400050084

[B19] Borgmeier T Estudos sobre *Atta *(Hym. Formicidae) Memórias do Instituto Oswaldo Cruz 1950 48 239 263 24539404 10.1590/s0074-02761950000100010

[B20] Hernández JV Jaffé K Economic damage caused by leaf-cutting ant populations of *Atta laevigata *(F. Smith) on pine plantations (*Pinus caribaeae *Mor.) and elements for managing of the pest An Soc Entomol 1995 24 287 298

[B21] Delabie JHC Vander Meer RK, Jaffe K, Cedeño A. Boulder The ant problems of cocoa farms in Brazil Applied myrmecology: a world perspective 1990 Colorado USA: Westview Press 555 569

[B22] Zanetti R Zanuncio JC Souza-Silva A Mendonça LA Mattos JOS Rizental MS Efficiency of products for thermonebulization on the control of *Atta laevigata *(Hymenoptera: Formicidae) in eucalypus plantations Ciênc Agrotec 2008 32 1313 1316 10.1590/S1413-7054200800040004321698667

[B23] Vasconcelos HL Cherret JM Leaf-cutting ants and early Forest regeneration in central Amazonia: effects of herbivory on the seedling establishment J Trop Ecol 1977 13 357 370

[B24] Viana LR Santos JC Arruda LJ Santos GP Fernandes GW Foraging patterns of the leaf-cutter ant *Atta laevigata *(Smith) (Myrmicinae: Attini) in an area of cerrado vegetation Neotrop Entomol 2004 33 391 393 10.1590/S1519-566X2004000300019

[B25] Michels K Cromme N Glatzle A Schultze-Kraft R Biological Control of Leaf-Cutting Ants Using Forage Grasses: Nest Characteristics and Fungus Growth J Agron Crop Sci 2001 187 259 267 10.1046/j.1439-037X.2001.00528.x

[B26] Ballari SA Farji-Brener AG Refuse dumps of leaf-cutting ants as a deterrent for ant herbivory: does refuse age matter? Entomol Exp Appl 2006 121 215 219 10.1111/j.1570-8703.2006.00475.x

[B27] Ying GG Kookana RS Persistence and movement of fipronil termiticide with under slab and trenching treatments Environ Toxicol Chem 2006 25 2045 2050 10.1897/05-652R.1 16916023

[B28] Ambrozin ARP Leite AC Bueno FC Vieira PC Fernandes JB Bueno OC Silva MFGF Pagnocca FC Hebling MJA Bacci M Limonoids from andiroba oil and *Cedrela fissilis *and their insecticidal activity J Braz Chem Soc 2006 17 542 547

[B29] Vettore AL Silva FR Kemper EL Arruda P The libraries that made SUCEST Genet Mol Biol 2001 24 1 7 10.1590/S1415-47572001000100002

[B30] Durham AM Kashiwabara AY Matsunaga FT Ahagon PH Rainone F Varuzza L Gruber A EGene: a configurable pipeline generation system for automated sequence analysis Bioinformatics 2005 21 2812 2813 10.1093/bioinformatics/bti424 15814554

[B31] Huang X Madan A CAP3: A DNA sequence assembly program Genome Res 1999 9 868 877 10.1101/gr.9.9.868 10508846 PMC310812

[B32] Conesa A Gotz S Garcia-Gomez JM Terol J Talon M Robles M Blast2GO: a universal tool for annotation, visualization and analysis in functional genomics research Bioinformatics 2005 21 3674 3676 10.1093/bioinformatics/bti610 16081474

[B33] Altschul SF Madden TL Schäffer AA Zhang J Zhang Z Miller W Lipman DJ Gapped BLAST and PSI-BLAST: a new generation of protein database search programs Nucleic Acids Res 1997 25 3389 3402 10.1093/nar/25.17.3389 9254694 PMC146917

[B34] Gene Ontology Consortium The Gene Ontology (GO) project in 2006 Nucleic Acids Res 2006 34 D322 326 16381878 10.1093/nar/gkj021 PMC1347384

[B35] Quevillon E Silventoinen V Pillai S Harte N Mulder N Apweiler R Lopez R InterProScan: protein domains identifier Nucleic Acids Res 2005 33 W116 120 10.1093/nar/gki442 15980438 PMC1160203

[B36] Zdobnov EM Apweiler R InterProScan - an integration platform for the signature-recognition methods in InterPro Bioinformatics 2001 17 847 848 10.1093/bioinformatics/17.9.847 11590104

[B37] Emanuelsson O Brunak S Heijne G Nielsen H Locating proteins in the cell using TargetP, SignalP, and related tools Nat Protoc 2007 2 953 971 10.1038/nprot.2007.131 17446895

[B38] Wang J Jemielity S Paolo U Wurm Y Gräff J Keller L An annotated cDNA library and microarray for large-scale gene-expression studies in the ant *Solenopsis invicta* Genome Biol 2007 8 R9 10.1186/gb-2007-8-1-r9 17224046 PMC1839134

[B39] The Honeybee Genome Sequencing Consortium Insights into social insects from the genome of the honeybee *Apis mellifera* Nature 2006 443 931 949 10.1038/nature05260 17073008 PMC2048586

[B40] The *Nasonia *Genome Working Group Functional and Evolutionary Insights from the Genomes of Three Parasitoid *Nasonia *Species Science 2010 327 343 348 10.1126/science.1178028 20075255 PMC2849982

[B41] Wagner A Energy costs constrain the evolution of gene expression J Exp Zool (Mol Dev Evol) 2007 308 322 324 10.1002/jez.b.21152 17262826

[B42] Zhu F Xu J Palli R Ferguson J Palli SR Ingested RNA interference for managing the populations of the Colorado potato beetle, *Leptinotarsa decemlineata* Pest Manag Sci 2011 67 175 182 10.1002/ps.2048 21061270

[B43] Barbosa AEAD Albuquerque EVS Silva MCM Souza DSL Oliveira-Neto OB Valencia A Rocha TL Grossi-de-Sa MF α-amylase inhibitor-1 gene from *Phaseolus vulgaris *expressed in *Coffea arabica *plants inhibits α-amylases from the coffee berry borer pest BMC Biotechnol 2010 10 44 10.1186/1472-6750-10-44 20565807 PMC2914071

[B44] Huvenne H Smagghe G Mechanisms of dsRNA uptake in insects and potential of RNAi for pest control: a review J Insect Physiol 2010 56 227 235 10.1016/j.jinsphys.2009.10.004 19837076

[B45] Ranson H Claudianos C Ortelli F Abgrall C Hemingway J Sharakhova MV Unger MF Collins FH Feyereisen R Evolution of supergene families associated with insecticide resistance Science 2002 298 179 181 10.1126/science.1076781 12364796

[B46] Hodgson E Kerkut GA, Gilbert LC Microsomal mono-oxygenases Comprehensive Insect Physiology Biochemistry and Pharmacology 1985 Oxford: Pergamon Press 647 712

[B47] Ketterer B Coles B Meyer DJ The role of glutathione in detoxication Environ Health Perspect 1983 49 59 69 6339228 10.1289/ehp.834959 PMC1569131

[B48] Ranson H Rossiter L Ortelli F Jensen B Wang X Roth CW Collins FH Hemingway J Identification of a novel class of insect glutathione S-transferases involved in resistance to DDT in the malaria vector *Anopheles gambiae* Biochem J 2001 359 295 304 10.1042/0264-6021:3590295 11583575 PMC1222147

[B49] Hatton PJ Cummins I Cole DJ Edwards R Glutathione transferases involved in herbicide detoxification in the leaves of *Setaria faberi *(giant toxtail) Physiol Plant 1999 105 9 16 10.1034/j.1399-3054.1999.105103.x

[B50] Hayes JD Pulford DJ The glutathione S-transferase supergene family: regulation of GST and the contribution of the isoenzymes to cancer chemoprotection and drug resistance Crit Rev Biochem Mol Biol 1995 30 445 600 10.3109/10409239509083491 8770536

[B51] Arca P Hardisson C Suarez J Purification of a glutathione S-transferase that mediates fosfomycin resistance in bacteria Antimicrob Agents Chemother 1997 34 844 848 10.1128/aac.34.5.844 PMC171703 2193621

[B52] Karlstrom RO Wilder LP Bastiani MJ Lachesin: an immunoglobulin superfamily protein whose expression correlates with neurogenesis in grasshopper embryos Development 1993 118 509 522 8223276 10.1242/dev.118.2.509

[B53] Harman D Aging: a theory based on free radical and radiation chemistry J Gerontol 1956 11 298 300 13332224 10.1093/geronj/11.3.298

[B54] Corona M Hughes KA Weaver DB Robinson GE Gene expression patterns associated with queen honey bee longevity Mech Ageing Dev 2005 126 1230 1238 10.1016/j.mad.2005.07.004 16139867

[B55] Spencer CC Howell CE Wright AR Promislow DE Testing an 'aging gene' in long-lived *Drosophila *strains: increased longevity depends on sex and genetic background Aging Cell 2003 2 123 130 10.1046/j.1474-9728.2003.00044.x 12882325 PMC3991309

[B56] Perez-Campo R Lopez-Torres M Cadenas S Rojas C Barja G The rate of free radical production as a determinant of the rate of aging: evidence from the comparative approach J Comp Physiol 1998 168 149 158 10.1007/s003600050131 9591361

[B57] Riddiford LM Cellular and molecular actions of juvenile hormone I. General considerations and premetamorphic actions Adv Insect Physiol 1994 24 213 274

[B58] Truman JW Riddiford LM The origins of insect metamorphosis Nature 1999 401 447 452 10.1038/46737 10519548

[B59] Wyatt GR Davey KG Cellular and molecular action of juvenile hormone II. Roles of juvenile hormone in adult insects Adv Insect Physiol 1996 26 1 155

[B60] Gillespie JP Kanost MR Trenczed T Biological mediators of insect immunity Annu Rev Entomol 1997 42 611 643 10.1146/annurev.ento.42.1.611 9017902

[B61] Ferrandon D Imler JL Hetru C Hoffmann JA The *Drosophila *systemic immune response: sensing and signalling during bacterial and fungal infections Nat Rev Immunol 2007 7 862 874 10.1038/nri2194 17948019

[B62] Nichol H Law JH Winzerling JJ Iron metabolism in insects Annu Rev Entomol 2002 47 535 559 10.1146/annurev.ento.47.091201.145237 11729084

[B63] Rodrigues A Bacci M Mueller UG Ortiz A Pagnocca FC Microfungal Weeds in the Leafcutter Ant Symbiosis Microb Ecol 2008 56 604 614 10.1007/s00248-008-9380-0 18369523

[B64] Poulin R Morand S Parasite biodiversity 2004 Washington: Smithsonian Books

[B65] Jackson DE Ratnieks FLW Communication in ants Curr Biol 2006 16 570 574 10.1016/j.cub.2006.01.064 16890508

[B66] Vogt RG Prestwich GD, Blomquist GJ The molecular basis of pheromone reception: its influence on behavior Pheromone Biochemistry 1987 New York: Academic Press 385 431

[B67] Krieger MJB Ross KG Identification of a Major Gene Regulating Complex Social Behavior Science 2002 295 328 332 10.1126/science.1065247 11711637

[B68] Kamikouchi A Morioka M Kubo T Identification of honeybee antennal proteins/genes expressed in a sex- and/or caste selective manner Zool Sci 2004 21 53 62 10.2108/0289-0003(2004)21[53:IOHAGE]2.0.CO;2 14745104

[B69] Flower DR The lipocalin protein family: structure and function Biochem J 1996 318 1 14 8761444 10.1042/bj3180001 PMC1217580

[B70] Ozaki M Wada-Katsumata A Fujikawa K Iwasaki M Yokohari F Satoji Y Nisimura T Yamaoka R Ant nestmate and non-nestmate discrimination by a chemosensory sensillum Science 2005 309 311 314 10.1126/science.1105244 15947139

[B71] Forêt S Wanner KW Maleszka R Chemosensory proteins in the honey bee: Insights from the annotated genome, comparative analysis and expressional profiling Insect Biochem Mol Biol 2007 37 19 28 10.1016/j.ibmb.2006.09.009 17175443

[B72] Todres E Nardi JB Robertson HM The tetraspanin superfamily in insects Insect Mol Biol 2000 9 581 590 10.1046/j.1365-2583.2000.00222.x 11122467

[B73] Maecker HT Todd SC Levy S The tetraspanin superfamily: molecular facilitators FASEB J 1997 11 428 442 9194523

[B74] Thany SH Crozatier M Raymond-Delpech V Gauthier M Lenaers G Apisα2, Apisα7-1 and Apisα7-2: Three new neuronal nicotinic acetylcholine receptor α-subunits in the honeybee brain Gene 2005 344 125 132 15656979 10.1016/j.gene.2004.09.010

[B75] Matsuda K Buckingham SD Kleier D Rauh JJ Grauso M Sattelle DB Neonicotinoids: Insecticides acting on insect nicotinic acetylcholine receptors Trends Pharmacol Sci 2001 22 573 580 10.1016/S0165-6147(00)01820-4 11698101

[B76] Siqueira CG Bacci M Pagnocca FC Bueno OA Hebling MJA Metabolism of plant polysaccharies by Leucoagariccus gongylophorus, the symbiotic fungus of the ant *Atta sexdens *L Appl Environ Microbiol 1998 64 4820 4822 9835568 10.1128/aem.64.12.4820-4822.1998 PMC90928

[B77] Silva A Bacci M Siqueira CG Bueno OC Pagnocca FC Hebling MJA Survival of *Atta sexdens *workers on different food sources J Insect Physiol 2003 49 307 313 10.1016/S0022-1910(03)00004-0 12769984

[B78] Zhou G Somasundaram T Blanc E Parthasarathy G Ellington WR Chapman MS Transition state struture of arginine kinase: implications for catalysis of biomolecular reactions Proc Natl Acad Sci USA 1998 95 8449 8454 10.1073/pnas.95.15.8449 9671698 PMC21096

[B79] Brown AE Grossman SH The mechanism and modes of inhibition of arginine kinase from the cockroach (Periplaneta americana) Arch Insect Biochem Physiol 2004 57 166 177 10.1002/arch.20026 15540275

